# Bifurcation analyses and potential landscapes of a cortex–basal ganglia–thalamus model

**DOI:** 10.1049/syb2.12018

**Published:** 2021-04-16

**Authors:** Chenri Yan, Quansheng Liu, Yuanhong Bi

**Affiliations:** ^1^ School of Mathematical Sciences Inner Mongolia University Hohhot China; ^2^ School of Statistics and Mathematics Inner Mongolia University of Finance and Economics Hohhot China

## Abstract

The dynamics of cortical neuronal activity plays important roles in controlling body movement and is regulated by connection weights between neurons in a cortex–basal ganglia–thalamus (BGCT) loop. Beta‐band oscillation of cortical activity is closely associated with the movement disorder of Parkinson's disease, which is caused by an imbalance in the connection weights of direct and indirect pathways in the BGCT loop. In this study, the authors investigate how the dynamics of cortical activity are modulated by connection weights of direct and indirect pathways in the BGCT loop under low dopamine levels through bifurcation analyses and potential landscapes. The results reveal that cortical activity displays rich dynamics under different connection weights, including one, two, or three stable steady states, one or two stable limit cycles, and the coexistence of one stable limit cycle with one stable steady state or two stable ones. For a low dopamine level, cortical activity exhibits oscillation for larger connection weights of direct and indirect pathways. The stability of these stable dynamics is explored by the potential landscapes.

## 1 INTRODUCTION

Neuronal activity in cortex plays a central role in motor dysfunction of Parkinson's disease [1]. Decreased firing rate or oscillatory activity in cortical neurons are frequently observed in patients with Parkinson's disease [2, 3]. Many models have investigated the relationship between cortical neuronal activity in the cortex–basal ganglia–thalamus (BGCT) loop and motor abnormalities in Parkinson's disease [4, 5]. Classical firing rate models hold that motor dysfunction in Parkinson's disease is attributed to the change in the firing rate of neurons in the BGCT loop, while recent electrophysiological studies show that these movement disorder are closely associated with abnormal firing patterns such as oscillation and neuronal synchronisation in the BGCT loop [6, 7]. Especially, oscillation in the beta frequency (13–30 Hz) is the main neuronal dynamical feature of Parkinson's disease, which has attracted more researchers to explore the source of beta‐band oscillation in the BGCT loop through mathematical models [8].

A series of computational models have been constructed to investigate the source of beta‐band oscillation and explore pathological behaviours of Parkinson's disease [9, 10, 11]. In these models, the negative feedback loop between subthalamic nucleus (STN) and the external segment of globus pallidus (GPe) has been considered to be the main reason for the generation of pathological beta‐band oscillations [12, 13]. However, abnormal synchronous oscillations were observed not only in STN‐GPe, but also in cortex, thalamus, and globus pallidus [14, 15]. Therefore, inputs from other regions, such as the cortex, to the basal ganglia, are also necessary to generate pathological oscillating activity. Cortex plays an important role in generating oscillation through a superdirect loop, where the cortex sends input to the STN, and receives indirect feedback from the STN and GPe via the internal segment of the globus pallidus (GPi) and thalamus [16]. However, an imbalance of connection weights from striatum to GPi and GPe is a critical source of beta‐band oscillation [17, 18, 19]. This imbalance is aroused by a low dopamine level, which decreases the excitation to striatal neurons with D1 receptors in the direct pathway and the inhibition of striatal neurons with D2 receptors in the indirect pathway [2]. These changes increase the neuronal activity of Gpi/SNr, which leads to decreased activity in the thalamus and cortical neurons and movement disorders. Nevertheless, little is known about beta‐band oscillation of neuronal activity in the cortex that occur upon different connection weights of the direct and indirect pathways at low dopamine levels.

In this work, based on a cortex–basal ganglia–thalamus loop regulated by dopamine [20, 21, 22, 23], the authors investigate how the dynamics of neuronal activity in the cortex depend on connection weights of direct and indirect pathways in the BGCT loop through bifurcation analyses and potential landscapes. A global bifurcation analysis reveals that neuronal activity in the cortex display rich dynamics, including monostability, bistability with two stable steady states or two stable limit cycles, tristability with three stable steady states, an oscillatory state, and the coexistence of oscillation with a stable steady state or two stable ones. The stability of these dynamics was further investigated through potential landscapes. The model is described in Section2, the results are analysed in Section 3, and finally the discussion is presented in Section4.

## 2 DESCRIPTION OF THE MODEL

**FIGURE 1:**
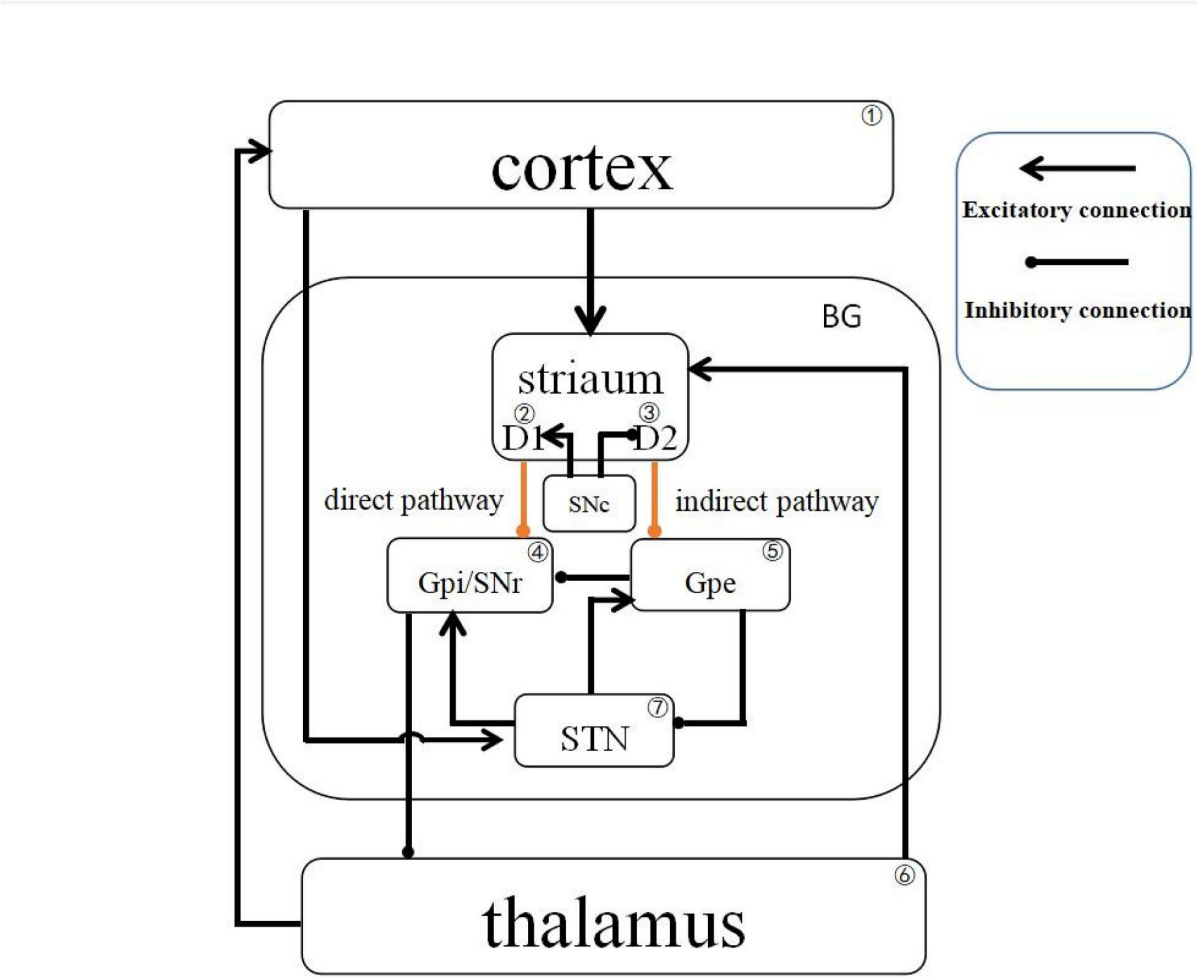
An illustration of the cortex–basal ganglia–thalamus loop regulated by dopamine from the substantia nigra pars compacta (SNc). Three loops are included, that is, the superdirect loop of Ctx‐STN‐GPi‐TH‐Ctx, the direct loop of Ctx‐D1‐GPi‐TH‐Ctx, and the indirect loop of Ctx‐D2‐GPe‐STN‐GPi‐TH‐Ctx. Arrow‐headed and solid‐circle‐headed solid lines represent excitatory and inhibitory connections, respectively

Figure 1 shows the cortex (Ctx)–basal ganglia (BG)–thalamus (TH) model proposed in [22]. This model consists of seven neuronal populations: cortex, striatum with D1 (D1) and D2 (D2) receptors, the internal (GPi) and external (GPe) segments of the globus pallidus, thalamus (TH), and subthalamic nucleus (STN), which are labelled 1–7, respectively. The arrows and solid point circles, respectively, denote excitatory and inhibitory synapses between neuronal populations. In the model, the cortex projects excitatory glutamatergic neurotransmitters to the STN and striatum, where a superdirect loop is formed from the Ctx to STN via GPe, GPi, and TH. Striatum as the input part of the basal ganglia is composed of medium spine neurons (MSNs) with D1 and D2 receptors, which send inhibitory aminobutyric acid neurotransmitters to GPi and GPe through direct and indirect pathways, respectively. In the indirect pathway, GPe forms inhibitory synapses with GPi and STN, and STN provides excitatory connections to GPi and GPe, where a negative feedback loop is formed between GPe and STN. Therefore, the direct and indirect pathways in the BGCT loop exert opposite roles on the activity of GPi. GPi as the output part of the basal ganglia suppresses neuronal activity in the thalamus, which further activates neuronal activity in the cortex and striatum. Moreover, dopamine in substantia nigra pars compacta (SNc) activates and inhibits the direct and indirect pathways through D1 and D2 receptors of MSNs in the striatum, respectively. However, dopamine depletion in Parkinson's disease reduces the connection weight in the direct pathway and increases it in the indirect pathway.

The dynamics of the above model can be quantitatively described by Equations (1), (2), (3), (4), (5), (6), (7), where *x*_*i*_ represents the activity of the corresponding neuronal population and *D*_input_ is the dopamine level. The response function in Equations (1), (2), (3), (4), (5), (6), (7) is the Hill function, where *s* and *n* are the Hill coefficient and index, respectively [24]. *T*_*ij*_ denotes the connection weight from neuronal population *j* to *i*, ranging between 0 and 7 [25]. *C*_*i*_ and *R*_*i*_ are the membrane capacitance and resistance. *τ* = *RC* = 6 ms means the time constant, and the corresponding parameter value *C*_*i*_ is expressed as *C*_*i*_ = *τ*/*R*_*i*_ [26]. *I*_*i*_ is the external input. The specific parameter values are shown in Table 1. Here, *T*_42_ and *T*_53_ as connection weights of direct and indirect pathways from striatum to GPi and GPe, are key parameters to be considered in the following sections.

(1) $$\begin{array}{ccc} \frac{dx_{1}}{dt} & = & \frac{1}{C_{1}}\left(I_{1}-\frac{x_{1}}{R_{1}}+\frac{T_{16}*X_{6}^{n}}{s^{n}+x_{6}^{n}}\right) \end{array}$$

(2) $$\begin{array}{ccc} \frac{dx_{2}}{dt} & = & \frac{1}{C_{2}}\left(I_{2}-\frac{x_{2}}{R_{2}}+\frac{T_{21}*x_{1}^{n}}{s^{n}+x_{1}^{n}}+\frac{T_{26}*x_{6}^{n}}{s^{n}+x_{6}^{n}}+D_{input}\right) \end{array}$$

(3) $$\begin{array}{ccc} \frac{dx_{3}}{dt} & = & \frac{1}{C_{3}}\left(I_{3}-\frac{x_{3}}{R_{3}}+\frac{T_{31}*x_{1}^{n}}{s^{n}+x_{1}^{n}}+\frac{T_{36}*x_{6}^{n}}{s^{n}+x_{6}^{n}}-D_{input}\right) \end{array}$$

(4) $$\begin{array}{ccc} \frac{dx_{4}}{dt} & = & \frac{1}{C_{4}}\left(I_{4}-\frac{x_{4}}{R_{4}}+\frac{T_{47}*x_{7}^{n}}{s^{n}+x_{7}^{n}}-\frac{T_{42}*x_{2}^{n}}{s^{n}+x_{2}^{n}}-\frac{T_{45}*x_{5}^{n}}{s^{n}+x_{5}^{n}}\right) \end{array}$$

(5) $$\begin{array}{ccc} \frac{dx_{5}}{dt} & = & \frac{1}{C_{5}}\left(I_{5}-\frac{x_{5}}{R_{5}}+\frac{T_{57}*x_{7}^{n}}{s^{n}+x_{7}^{n}}-\frac{T_{53}*x_{3}^{n}}{s^{n}+x_{3}^{n}}\right) \end{array}$$

(6) $$\begin{array}{ccc} \frac{dx_{6}}{dt} & = & \frac{1}{C_{6}}\left(I_{6}-\frac{x_{6}}{R_{6}}-\frac{T_{64}*x_{4}^{n}}{s^{n}+x_{4}^{n}}\right) \end{array}$$

(7) $$\begin{array}{ccc} \frac{dx_{7}}{dt} & = & \frac{1}{C_{7}}\left(I_{7}-\frac{x_{7}}{R_{7}}+\frac{T_{71}*x_{1}^{n}}{s^{n}+x_{1}^{n}}-\frac{T_{75}*x_{5}^{n}}{s^{n}+x_{5}^{n}}\right) \end{array}$$

**TABLE 1:** Typical parameter values [22]

Parameter	Value	Parameter	**Value**
*C*_*i*_(*i* = 1, …, 7)	3.60	*R*_*i*_(*i* = 1, …, 7)	1.67
*τ*	6	*T*_16_	2
*T*_21_	1.4	*T*_26_	1.4
*T*_31_	1.4	*T*_36_	1.4
*T*_45_	3	*T*_47_	2
*T*_57_	1	*T*_64_	3.2
*T*_71_	1.8	*T*_75_	1.8
*s*	2	*n*	2
*D*_input_	0.6	*I*_1_	0.1
*I*_2_	0.05	*I*_3_	1.2
*I*_4_	4.4	*I*_5_	2.8
*I*_6_	2	*I*_7_	1.2

## 3 RESULTS

As a result of the decrease in the dopamine level, the connection weights between the striatum with Gpi/SNr in the direct pathway and Gpe in the indirect pathway change. To investigate how connection weights of direct and indirect pathways (*T*_42_ and *T*_53_) regulate neuronal activity in the cortex, the authors performed bifurcation analyses with respect to *T*_42_ and *T*_53_, ranging from 0 to 7. First a codimension‐one bifurcation diagram of *T*_42_ at typical *T*_53_ value is given, then codimension‐two bifurcation analysis of *T*_42_ and *T*_53_ is carried out, and typical time series and phase diagrams are drawn. Furthermore, the stability of these stable dynamics is explored by potential landscapes. In addition, a codimension‐two bifurcation diagram of the parameters *T*_45_ and *T*_47_, which character the connection weights between GPi with STN and GPe, is illustrated. All the figures are obtained by AUTO in XPPAUT [27] and Matlab.

### 3.1 Codimension‐one bifurcation analysis of *T*_42_

**FIGURE 2:**
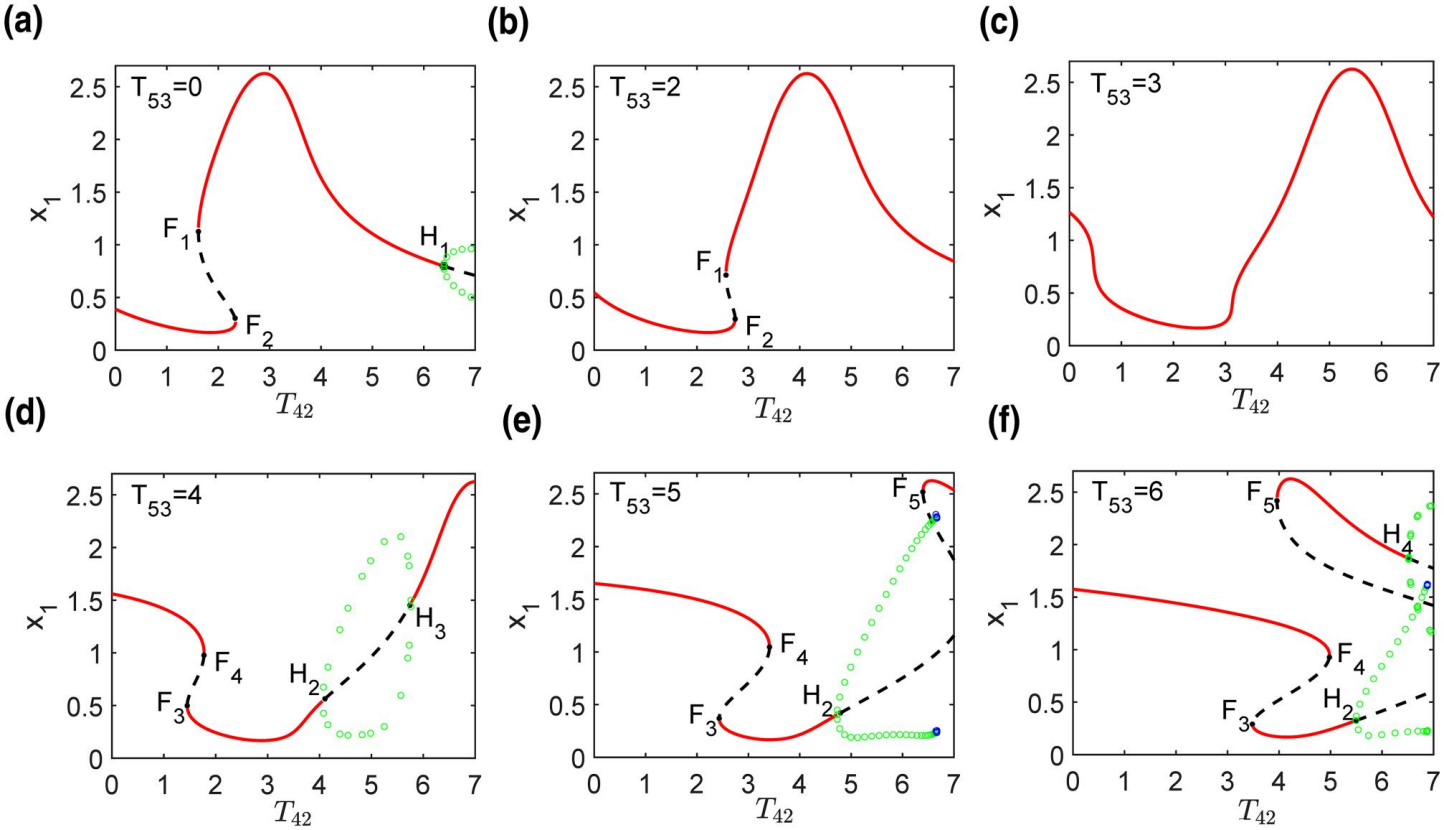
(a)–(f) Typical codimension‐one bifurcation diagrams of cortical activity versus *T*_42_ at given *T*_53_. In all diagrams, red solid lines and black dashed lines represent stable and unstable equilibria, respectively. The green open circles are the maxima and minima of stable limit cycles. *F*_*i*_(*i* = 1–5) are the fold bifurcation points of the equilibria. *H*_*i*_(*i* = 1–4) represents the Hopf bifurcation points

In this section, codimension‐one bifurcation of cortical activity (*x*_1_) is carried out with respect to *T*_42_ at typical *T*_53_ = 0, 2, 3, 4, 5, 6, as shown in Figure 2. In all diagrams, red solid lines and black dashed lines are stable nodes and unstable saddles, respectively. The green open circles represent the maxima and minima of stable limit cycles. *F*_*i*_(*i* = 1–5) and *H*_*i*_(*i* = 1–4) are fold bifurcation points of the equilibria and Hopf bifurcation points, respectively. The details of each diagram are as follows:

(1) At small *T*_53_ = 0, the upper and lower branches of the bifurcation diagram, respectively, meet at *F*_1_ and *F*_2_ with the middle branch, where two stable steady states exist between *F*_1_ and *F*_2_. Also, a supercritical Hopf bifurcation point *H*_1_ appears at larger *T*_42_ in the upper branch such that the stable steady state loses stability and is surrounded by a stable limit cycle.
(2) When *T*_53_ increases (*T*_53_ = 2), the Hopf bifurcation point *H*_1_ disappears so that only a stable steady state is left on the upper branch.
(3) With the further increase of *T*_53_ (*T*_53_ = 3), only one stable steady state is left due to the disappearance of *F*_1_ and *F*_2_.
(4) When *T*_53_ increases to 4, the bifurcation diagram is a Z‐shaped curve, where the bistable region of equilibria arises again between *F*_3_ and *F*_4_. Moreover, there are two supercritical Hopf bifurcation points *H*_2_ and *H*_3_ on the lower branch, where a stable limit cycle surrounds an unstable steady state between them.
(5) With the further increase of *T*_53_ (*T*_53_ = 5), a stable steady state and an unstable one arise from another fold bifurcation point *F*_5_. Also, the stable limit cycle from *H*_2_ ends on the saddle.
(6) For larger *T*_53_, *T*_53_ = 6, *F*_5_ moves to the left of *F*_4_ so that three steady states coexist between *F*_5_ and *F*_4_. Also, a supercritical Hopf bifurcation point *H*_4_ appears on the upper branch from *F*_5_, where there are two stable limit cycles on the right of *H*_4_.

With low dopamine levels, dopamine deficiency reduces the excitability of neurons containing *D*1 receptors and the inhibition of neurons containing *D*2 receptors in the striatum. As a result, the inhibitions from the striatum on GPi/SNr and GPe decrease and increase, respectively, leading to the generation of beta oscillation. Codimension‐one bifurcation analysis shows that the increase of *T*_53_ results in an obvious oscillation in the cortical activity. The results of these experiments are consistent.

### 3.2 Codimension‐two bifurcation analysis of *T*_42_ and *T*_53_

**FIGURE 3:**
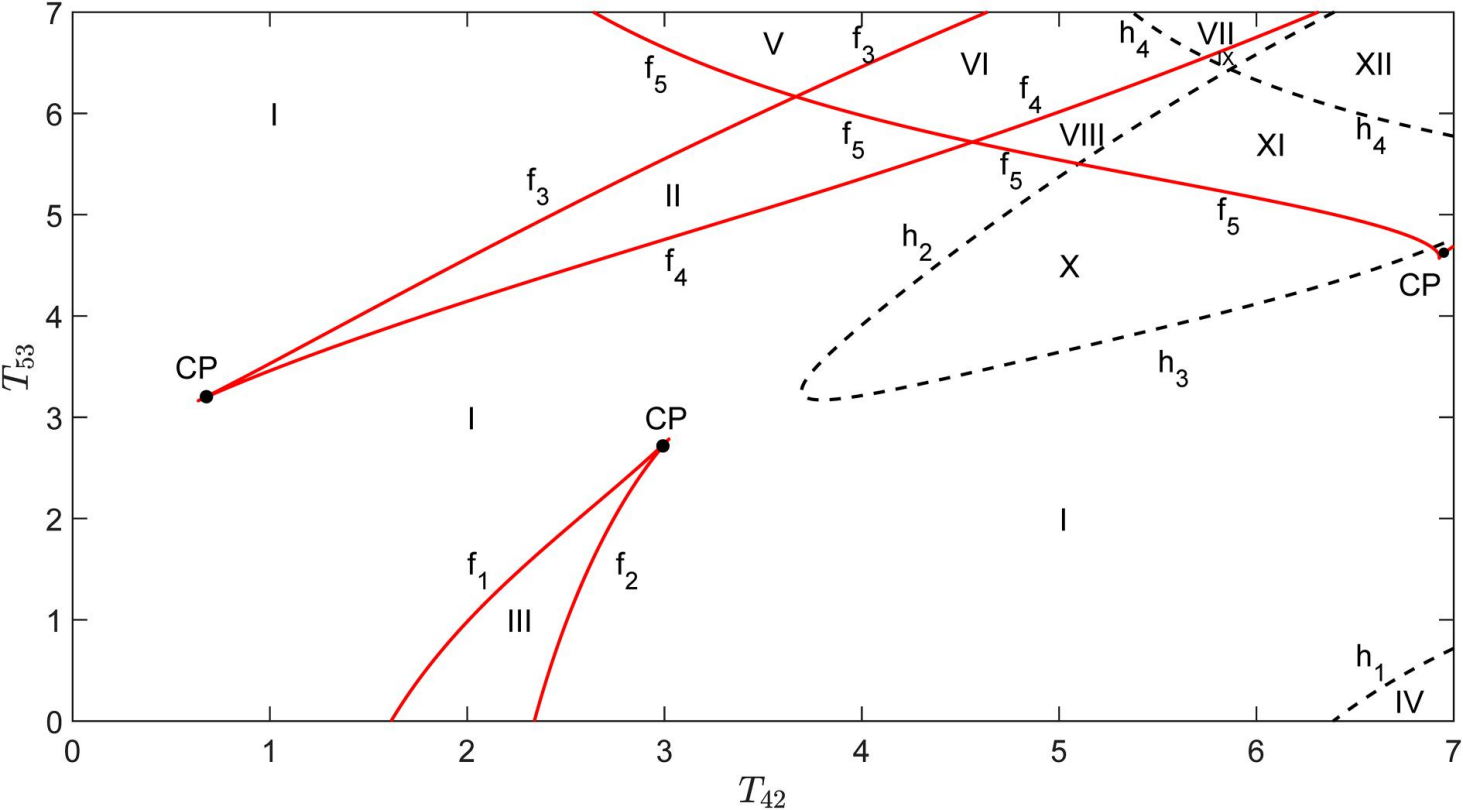
Codimension‐two bifurcation diagram with respect to the parameters *T*_42_ and *T*_53_. The parameter plane (*T*_42_, *T*_53_) is divided into 12 regions by codimension‐one bifurcation curves *f*_*i*_(*i* = 1–5) and *h*_*i*_(*i* = 1–4). *f*_*i*_ is the fold bifurcation point of the equilibria, *h*_*i*_ is the supercritical Hopf bifurcation point, and *CP*_*i*_ is the Cusp point of codimension‐two bifurcation

In order to give a more comprehensive view of the dynamics of cortical activity regulated by *T*_42_ and *T*_53_, codimension‐two bifurcation analysis of these two parameters is carried out in Figure 3. The parameter plane (*T*_42_, *T*_53_) is divided into 12 regions that are separated by red solid lines *f*_*i*_(*i* = 1–5) with fold bifurcation points of equilibria and black dashed lines *h*_*i*_(*i* = 1 ∼ 4) with supercritical Hopf bifurcation points. *f*_*i*_ bifurcates from codimension‐two Cusp bifurcation points *CP*_*i*_. The different dynamics in each region are described as follows:

(1) A stable steady state exists in region I.
(2) When the parameters in region I cross the curves *f*_1_, *f*_3_, and *f*_5_ and enter regions III, II, and V, respectively, another stable steady state (node) and an unstable one (saddle) appear due to fold bifurcation of equilibria such that there are two stable steady states and one unstable one in regions II, III, and V. Also, supercritical Hopf bifurcation *h*_1_ makes a stable steady state in region I unstable and a stable limit cycle appears such that only one stable limit cycle is left with an unstable steady state in region IV.
(3) Region VI can be reached from regions V or II through passing *f*_3_ or *f*_5_, which causes the appearance of a stable steady state and an unstable one. Therefore, three stable steady states and two unstable ones coexist in region VI.
(4) When the curve *h*_4_ is crossed from regions VI to VII, a stable steady state becomes unstable and gives rise to a stable limit cycle such that two stable steady states and a stable limit cycle coexist with three unstable steady states in region VII.
(5) Regions VIII and IX, respectively, are located below regions VI and VII and reach through the curve *f*_4_, which causes the disappearance of a stable steady state and an unstable one in regions VI and VII. Hence, two stable steady states and one unstable one are left in region VIII, while one stable steady state and two unstable ones coexist with one stable limit cycle in region IX.
(6) In region X, a stable limit cycle surrounds an unstable steady state when parameters enter region X through *h*_2_ or *h*_3_ from region I.
(7) One stable steady state and two unstable ones stay with one stable limit cycle in region XI due to the fold bifurcation curve *f*_5_.
(8) Region XII includes two stable limit cycles and three unstable steady states since the supercritical Hopf bifurcation curve *h*_4_ makes a stable limit cycle appear and a stable steady state unstable in region XI.

Through the codimension‐two bifurcation analysis, it can be clearly seen that only a steady‐state exists for smaller *T*_42_ and *T*_53_, and the oscillation will appear for larger *T*_42_ and *T*_53_, especially when *T*_53_ is greater than *T*_42_, which is consistent with the experimental results.

**FIGURE 4:**
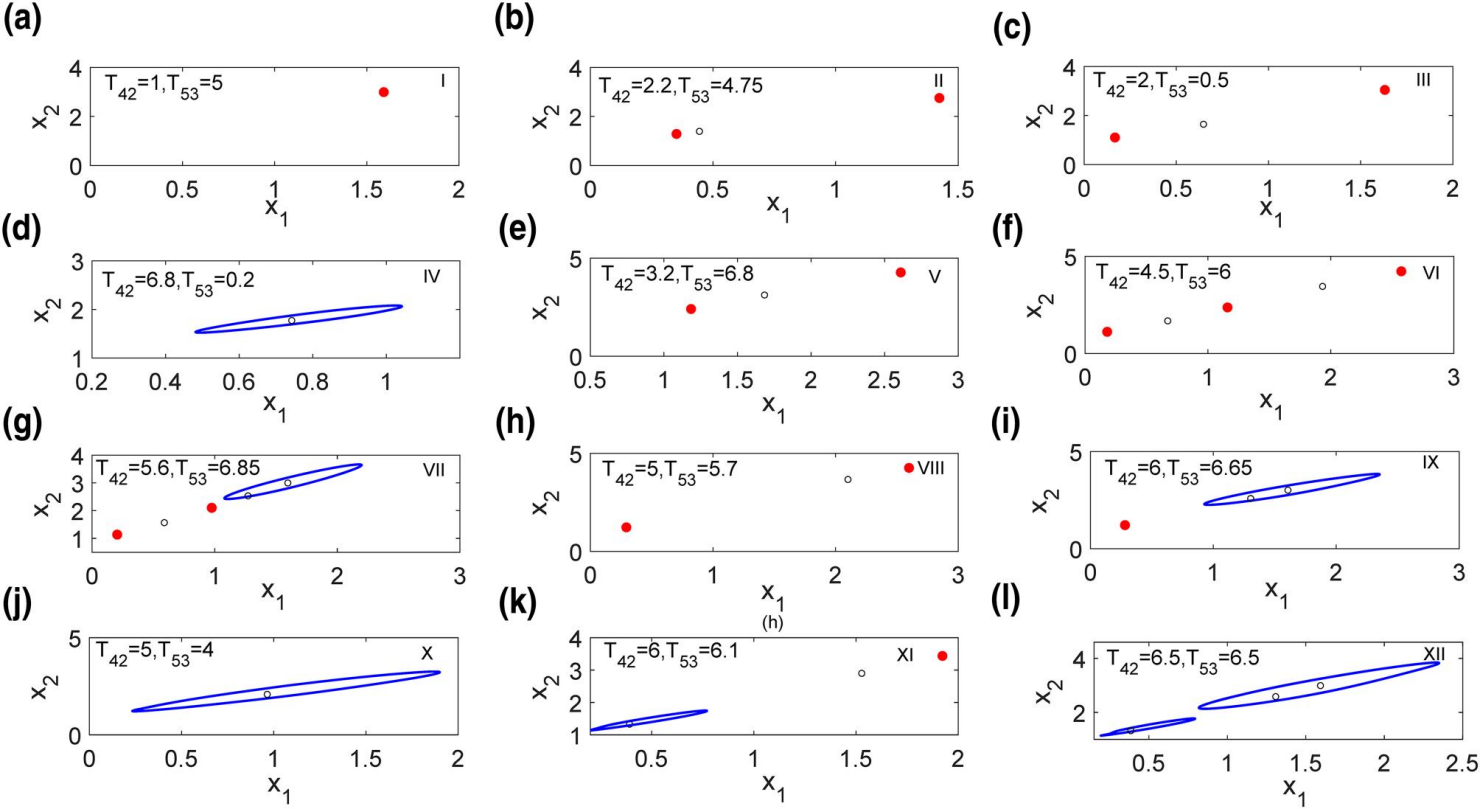
(a)–(l) Phase diagrams of regions I–XII in Figure 3. The red solid dots and black circles represent stable and unstable equilibria, respectively; The blue solid lines denote stable limit cycles

**FIGURE 5:**
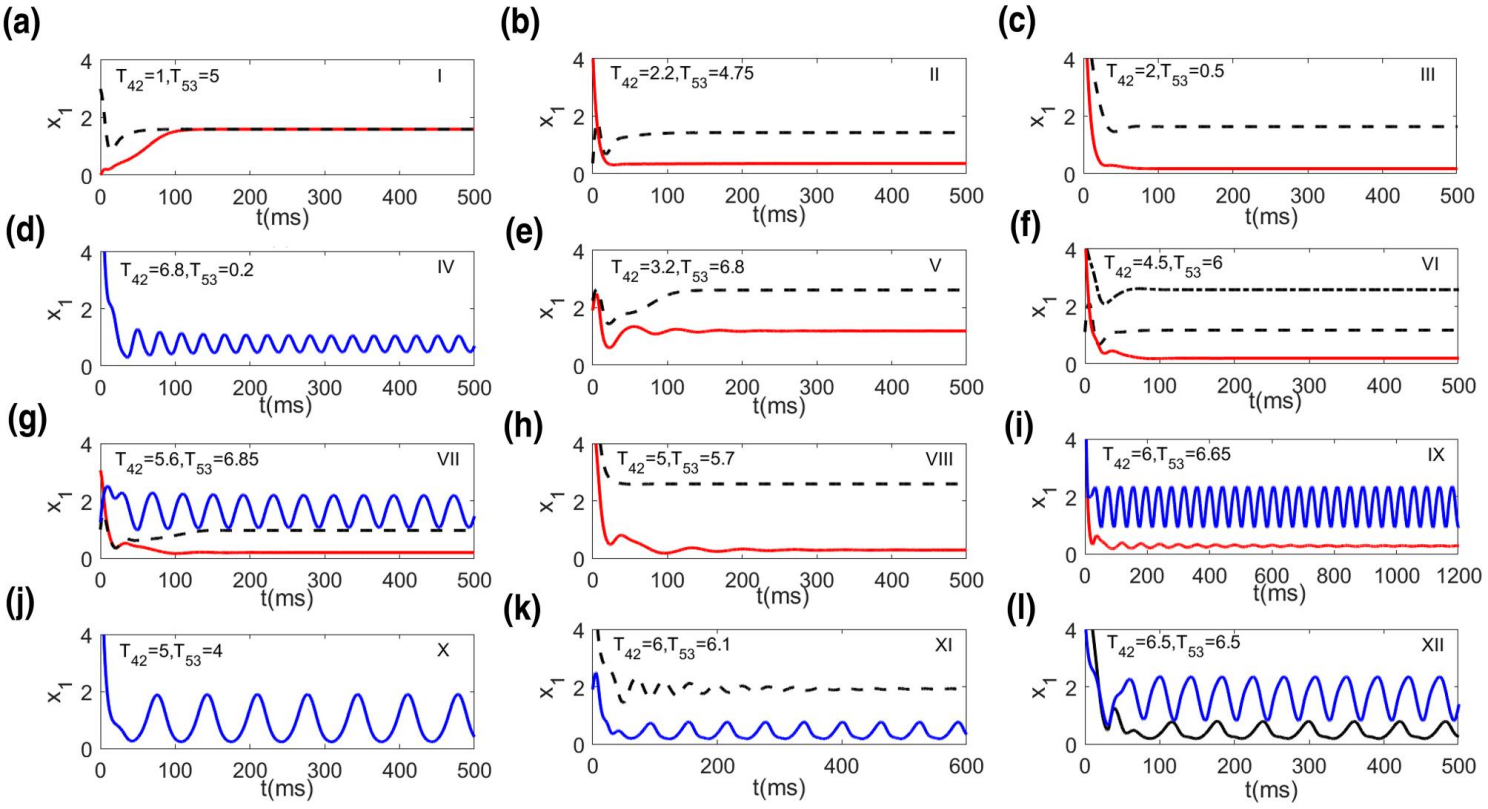
(a)–(l) Typical time series of cortical activity in regions I–XII of Figure 3. Stable dynamics in each region are given through changing initial values. Parameters *T*_42_ and *T*_53_ are the same as those in Figure 4

Furthermore, all the steady states in each region of Figure 3 are given in phase diagrams in Figure 4, where red solid dots are stable steady states and black open circles are unstable ones, and blue solid lines represent stable limit cycle. In summary, following different connection weights of direct and indirect pathways, *T*_42_ and *T*_53_, the dynamics of neuronal activity in cortex displays the following eight types of stable dynamics: a stable steady state (region I), two stable steady states (regions II, III, V, and VIII), a stable limit cycle (regions IV and X), three stable steady states (region VI), one stable steady state and one stable limit cycle (regions IX and XI), two stable steady states and one stable limit cycle (region VII), and two stable limit cycle s(region XII). Figure 5 shows the time series of the stable dynamics of neuronal activity in the cortex in each region of Figure 3, which are obtained by solving Equations (1), (2), (3), (4), (5), (6), (7) for different initial values, and the parameters are the same as those in Figure 4. The results obtained were consistent with those of bifurcation analyses.

In addition, the global stability of these stable dynamics can be explored through potential landscapes.

### 3.3 The potential landscape

**FIGURE 6:**
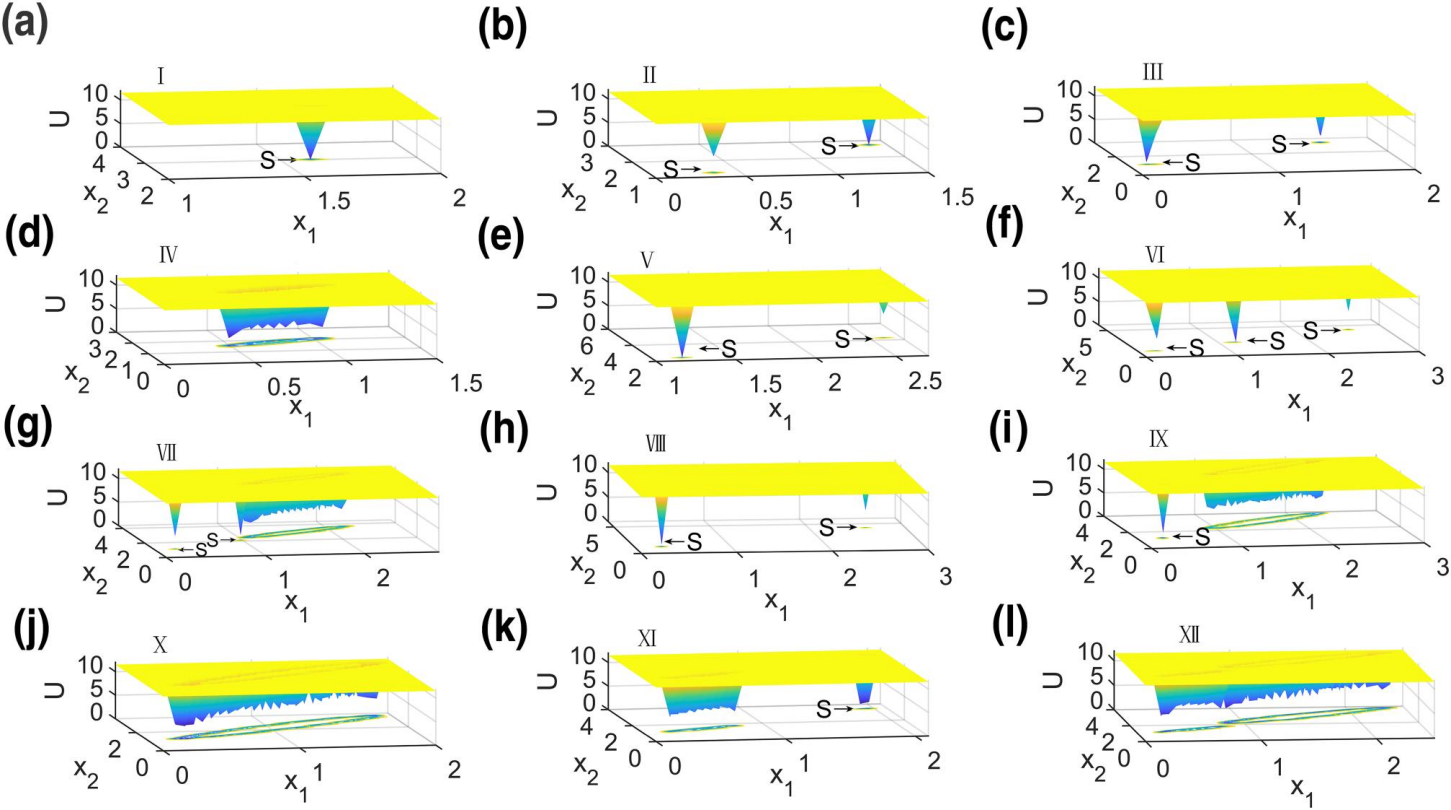
(a)–(l) The potential landscapes for 12 regions in Figure 3. Arrows with S point to the stable steady states, and irregular closed rings represent stable limit cycles. The parameters in each region are the same as those in Figure 4 and *D* = 1 × 10^−6^

The potential landscape *U* = −ln(*P*_*ss*_) proposed by Jin Wang can be used to explore the global stability of neuronal activity in cortex, where *P*_*ss*_ is the probability distribution of the stable steady states [28]. *U* = −ln(*P*_*ss*_) can be obtained by the following steps. Gaussian and white noise *ζ*(*t*) is firstly added into the right hand of Equations (1), (2), (3), (4), (5), (6), (7) to get stochastic differential equations, where < *ζ*(*t*)*ζ*(*t*′) > = 2*Dδ*(*t* − *t*′), < *ζ*(*t*) > = 0, and *D* is the correlation tensor (matrix) measuring the noise strength. Under different initial values, stochastic differential equations are simulated many times to achieve all stable steady states. Based on the distribution of these stable steady states, the probability distribution of stable steady states *P*_*ss*_ and then potential landscapes *U* = −ln(*P*_*ss*_) are obtained. Therefore, the least potential landscape means the largest probability and the highest stability of the stable steady state. *U* = −ln(*P*_*ss*_) is projected into the plane of *x*_1_ and *x*_2_ since *U* = −ln(*P*_*ss*_) related with all variables is difficult to view. Figure 6 shows all potential landscapes in each region of Figure 3, where parameters in each region are the same as those in Figure 4 and *D* = 1.0 ∗ 10^−6^. The global minimum of the potential landscape corresponds to the stable steady state, which is marked by S with an arrow. Based on Figure 6, a global minimum of the potential landscape corresponds to a stable steady state in region I. In regions II, III, V, and VIII, two local minima of the potential landscape mean two stable steady states with high and low neuronal activity, where the higher steady state is more stable than the low one in region II, while it is the opposite in regions III, V, and VIII. The middle steady state is the most stable in region VI according to three local minima of potential landscape. In regions IV, VII, IX, X, XI, and XII, the irregular and inhomogeneous closed ring means a stable limit cycle, while local minima in regions VII, IX, and XI show the coexistence of the stable steady state marked by S and the arrows.

The potential landscape can measure the global stability and coherent oscillation of the system well. As mentioned earlier, the output of the basal ganglia is determined by the balance between direct and indirect pathways. The direct pathway promotes movement, while the indirect pathway inhibits movement. In the above analysis, for the above 12 regions, the oscillation mainly occur when the connection weights of striatum‐GPi and striatum‐GPe are high. Especially when *T*_53_ is greater than *T*_42_ in regions VII, IX, and XI, there are two kinds of steady state, one is a funnel‐like steady state, and the other is a basin‐like oscillation, which can be realised through varying the initial conditions. During the treatment of Parkinson's disease, the abnormal oscillations in the cortex–basal ganglia–thalamus can be eliminated by deep brain stimulation, so that the neurons can fire healthily and stably, and the manifestations of Parkinson's disease can be inhibited. In Parkinson's disease, the oscillations were changed to a stable state by changing the external input to the relevant brain regions.

### 3.4 Codimension‐two bifurcation analysis of *T*_45_ and *T*_47_

For a more comprehensive understanding of the origin of oscillations, the influence of the connection weights of STN‐GPi and GPe‐GPi on cortical activity are considered. GPi acting as an output nucleus of the basal ganglia receives the inhibitory neurotransmitter GABA from GPe and the excitatory neurotransmitter glutamate from STN, and integrates signals from other brain regions to the thalamus and cortex. Under the condition of a low dopamine level, the inhibitory effect of GPe on GPi is weakened, and the excitability of STN on GPi is increased.

To investigate how cortical activity is regulated by *T*_45_ and *T*_47_, a codimension‐two bifurcation analysis of these two parameters is presented. The parameter plane (*T*_45_, *T*_47_) is divided into six regions by black dashed lines *h*_*i*_(*i* = 1∼2) with supercritical Hopf bifurcation points and red solid lines *f*_*i*_(*i* = 1∼6) with fold bifurcation points of equilibria. *CP*_*i*_ is the Cusp bifurcation point. The detailed description is as follows:

(1) Only a stable equilibrium point exists in region I.
(2) The parameters in region I cross the curves *f*_*i*_, (*i* = 1, …, 6) composed of fold bifurcation points, and enter regions II, III, and IV. *f*_*i*_ makes a new stable node and an unstable saddle appear so that two stable nodes and one unstable saddle exist in regions II, III, and IV.
(3) Region V can be reached from region I through the curves *h*_1_ and *h*_2_, which makes a stable equilibrium point become unstable and gives rise to a stable limit cycle. Only a stable limit cycle and an unstable steady state are left in region V.
(4) *f*_3_ locating between regions V and VI makes stable and unstable equilibrium points appear when parameters in region V pass through *f*_3_ into region VI. Therefore, there are a stable limit cycle, a stable equilibrium point, and two unstable equilibrium points in region VI.

**FIGURE 7:**
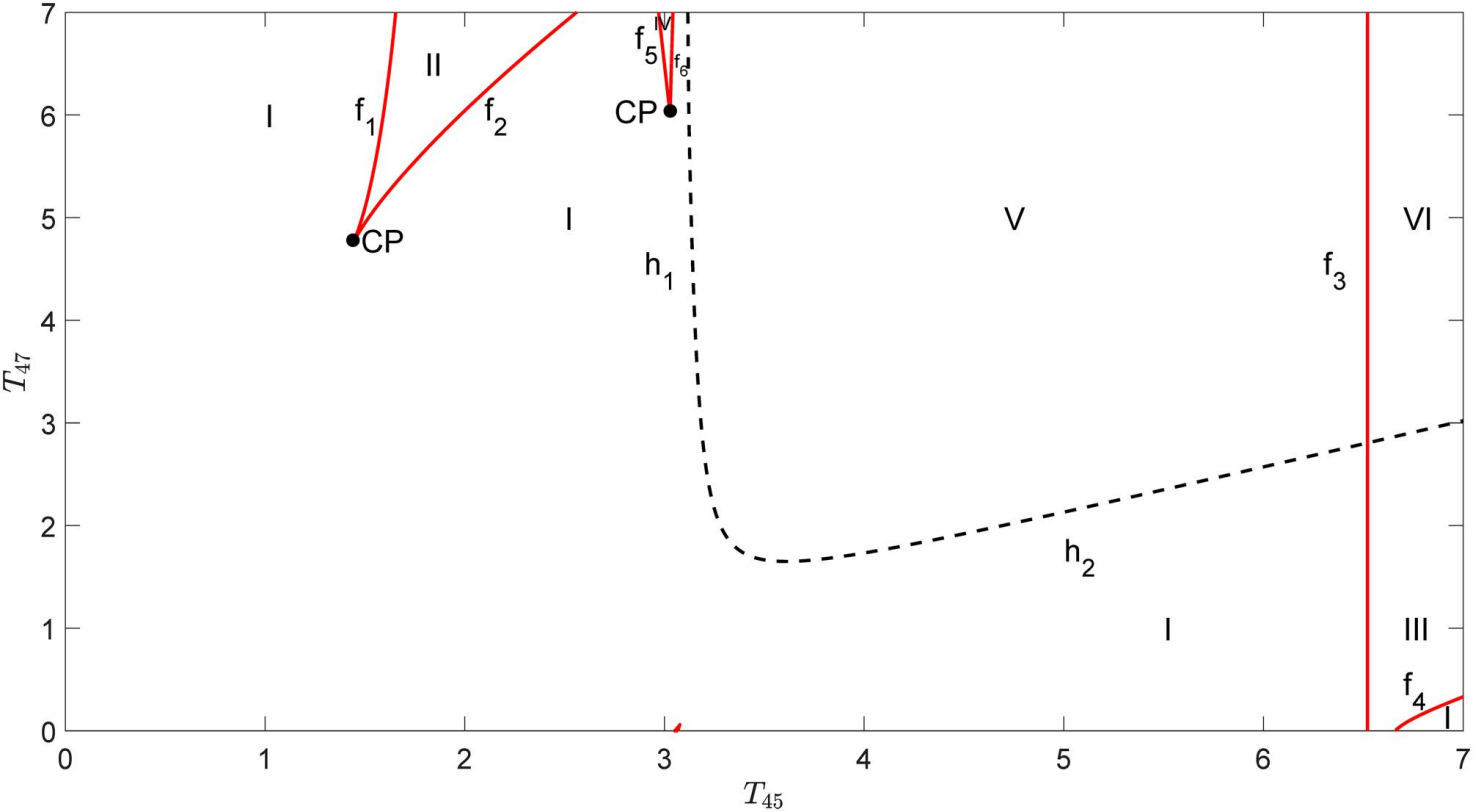
Codimension‐two bifurcation diagram with respect to the parameters *T*_45_ and *T*_47_. The parameter plane (*T*_45_, *T*_47_) is divided into six regions by curves *f*_*i*_(*i* = 1∼6) and *h*_*i*_(*i* = 1∼2). *f*_*i*_ is the fold bifurcation point of the equilibria, *h*_*i*_ is the supercritical Hopf bifurcation point, and *CP*_*i*_ is the Cusp point of codimension‐two bifurcation

In brief, six regions in Figure 7 show four stable dynamics: a stable steady state (region I), two stable steady states (regions II, III, and IV), a stable limit cycle (region V), and one stable steady state and one stable limit cycle (region VI). Therefore, cortex activity exhibits oscillation under certain *T*_45_ and *T*_47_.

## 4 DISCUSSION

The dynamics of neuronal activity in cortex plays an important role in controlling movement and is affected by connection weights between neurons. Connection weights in the direct and indirect pathways of the BGCT loop are affected by a low dopamine level in Parkinson's disease, where the low dopamine level leads to less activation of the direct pathway and more inhibition of the indirect pathway in the BGCT loop. Herein, the authors systematically study the effects of connection weights of direct and indirect pathways on neuronal activity in cortex, and the results reveal that under low dopamine, neuronal activity in the cortex shows monostability or bistability for either smaller *T*_42_ or smaller *T*_53_. When *T*_42_ and *T*_53_ increase, beta‐band oscillation of neuronal activity in the cortex emerges through Hopf bifurcation. Additionally, the coexistence of oscillation and stable states or two stable limit cycles are possible for proper *T*_42_ or *T*_53_. These results are further verified by time series and phase diagrams. With the decrease in dopamine level, the excitability of neurons containing D1 and D2 receptors in the striatum decrease and increase, respectively, leading to an increase in the direct pathway inhibitory input and a decrease in the indirect pathway excitability input. Through the above bifurcation analysis, it can be clearly observed that at low dopamine levels, the D2–GPe connection weight in the indirect pathway is greater than the D1–GPi connection weight in the direct pathway in a certain region, and there is an oscillation phenomenon. Also, the stability of these dynamics is explored by the potential landscapes. Through the analysis of the potential landscapes, the stability of different steady states can be seen and the coexistence of steady state and oscillation found. Medically, Parkinson's disease can be alleviated by deep brain stimulation in certain areas of the brain. Correspondingly, the initial values can be changed in the established model to make oscillatory activity become steady state. This also provides a theoretical basis for the treatment of Parkinson's disease. Finally, the codimension‐two bifurcation diagram of connection weights of STN‐GPi and GPe‐GPi is given to explore the influence of these two weights (*T*_45_ and *T*_47_) on cortical activity. These results highlight the importance of the connection weights of direct and indirect pathways.

Herein, the authors have focused on the BGCT loop regulated by dopamine and explores neuronal activity in the cortex regulated by connection weights of direct and indirect pathways. However, it is necessary to explore the effects of other connection weights and the time delays of synaptic connections between neurons on neuronal activity in this loop in future work.
